# Glioma-amplified sequence *KUB3* influences double-strand break repair after ionizing radiation

**DOI:** 10.3892/ijo.2013.1937

**Published:** 2013-05-13

**Authors:** ULRIKE FISCHER, STEFANIE RHEINHEIMER, ANDREA KREMPLER, MARKUS LÖBRICH, ECKART MEESE

**Affiliations:** 1 Department of Human Genetics, Medical School, Saarland University, D-66421 Homburg/Saar;; 2 Radiation Biology and DNA Repair, Darmstadt University of Technology, D-64287 Darmstadt, Germany

**Keywords:** gene amplification, radiation-resistance, glioblastoma

## Abstract

Human glioblastomas are characterized by frequent DNA amplifications most often at chromosome regions 7p11.2 and 12q13-15. Although amplification is a well-known hallmark of glioblastoma genetics the function of most amplified genes in glioblastoma biology is not understood. Previously, we cloned Ku70-binding protein 3 (KUB3) from the amplified domain at 12q13-15. Here, we report that glioblastoma cell cultures with endogenous *KUB3* gene amplification and with elevated KUB3 protein expression show an efficient double-strand break (DSB) repair after being irradiated with 1 Gy. A significantly less efficient DSB repair was found in glioma cell cultures without KUB3 amplification and expression. Furthermore, we found that a siRNA-mediated reduction of the endogenous KUB3 expression in glioblastoma cells resulted in a reduction of the repair efficiency. HeLa cells transfected with *KUB3* showed an increased DSB repair in comparison to untreated HeLa cells. In addition, KUB3 seems to influence DSB efficiency via the DNA-PK-dependent repair pathway as shown by simultaneous inhibition of KUB3 and DNA-PK. The data provide the first evidence for a link between the level of *KUB3* amplification and expression in glioma and DSB repair efficiency.

## Introduction

DNA amplification frequently occurs in human tumors but very rarely in normal human cells. Glioblastoma multiforme (GBM) that are characterized by a severe genomic instability show frequent amplifications predominantly as double minute chromosomes and less frequently as homogeneously staining region. DNA amplifications in GBM are found most often at 7p11.2 and 12q13-15 (1). Previously, we cloned several genes from the amplified domain at 12q13-15 including *GAS41* (glioma expressed sequence), *CYP27B1* and *GAS16* by microdissection-mediated cDNA capture (2–5). GAS16 results from an alternative splicing process of the gene *KUB3* coding for Ku70-binding protein 3 (KUB3). KUB3 was found to bind Ku70 by yeast two-hybrid screening (6). Ku70 has been associated with the DNA-dependent protein kinase (DNA-PK) complex involved in double-strand break repair. DNA double-strand breaks (DSBs) are considered to be the most relevant DNA lesions. Unrepaired DSBs can cause cell death and misrepaired DSBs may lead to chromosomal translocations and genomic instability that is a hallmark of GBM (7–9).

There are two major DSB repair pathways. The homologous recombination (HR) repairs the break using an undamaged homologous chromatid or chromosome, whereas the nonhomologous end-joining (NHEJ) ligates the DNA end directly (10). The main proteins required for NHEJ in mammalian cells are the DNA-dependent protein kinase (DNA-PK) formed by the Ku70/Ku80 heterodimer and the catalytic subunit DNA-PKcs in association with Artemis and the XRCC4/Ligase IV/Cernunnos-XLF complex. Ku and DNA-PKcs components are necessary to load this complex to the site of the break (11,12), whereas the XRCC4/Ligase IV complex is responsible for the ligation step (13,14).

Analyzing >100 primary glioma we found *KUB3* also termed *XRCC6BP1* (X-ray repair cross-complementation group 6 binding protein 1) to be amplified in 14% of GBM, 30% of anaplastic astrocytoma and 3% of pilocytic astrocytoma. Northern blot analysis of GBM showed a correlation between KUB3 amplification and overexpression. Amplification of *KUB3* was associated with significantly shorter survival time as shown by our experimental data and by *in silico* analysis of 185 GBM samples from the TCGA data collection (15).

With the frequent amplification of *KUB3* in glioma, especially in higher-grade glioma and with its association to DNA repair pathways, overexpression of KUB3 likely confers properties to growth advantage in GBM cells. This idea is consistent with the prolonged survival of patients with glioma without *KUB3* amplification in comparison to patients with *KUB3* amplification. Here, we set out to analyze the interrelationship between *KUB3* amplification, KUB3 expression and DSB-repair in glioma. Specifically, we address the following questions. Do glioma with a higher *KUB3 (XRCC6BP1)* amplification show a more efficient DSB repair than glioma with a lesser or none *KUB3* amplification? Does the endogenous expression level of KUB3 correlate with the DSB repair efficiency in glioma? Does induced reduction or induced increase of KUB3 expression affect the DSB repair efficiency? Does KUB3 act in a DNA-PK dependent manner? Overall, the study aims to contribute to understanding the cellular effects of *KUB3* amplification in human glioma.

## Materials and methods

### Ethics statement

Glioblastoma tissue samples were obtained from the Neurosurgery Department of Saarland University (Homburg, Germany) with patient’s written informed consent. The study was approved by the local ethics committee of the ‘Ärztekammer des Saarlandes’ on September 2001 for glioma study (Ethik-Komm./Ls).

### Cells and cell lines

Normal human astrocytes were obtained from CellSystems and maintained in ABM (astrocyte basal medium) without L-glutamine (Clonetics, Cambrex Bio Science, Walkersville, MD, USA). The glioblastoma cell lines TX3868 and TX3095 were established at our institute after xenografting. The glioblastoma cell cultures H346 and H385 and the astrocytoma cell culture T6468 were established at our institute. The different glioma cell lines and cultures were grown in DMEM supplemented with 10% fetal calf serum (PAA), 100 U/ml of penicillin and 100 *μ*g/ml of streptavidin (2,16,17). HeLa cells (ATCC) were maintained in DMEM containing 10% fetal calf serum (Biochrom AG, Berlin, Germany).

### Brain tumor samples

Tissues of 15 glioblastoma multiforme (WHO grade IV) were obtained from the Neurosurgery Department of Saarland University (Homburg, Germany) with patient’s written informed consent. The samples were frozen in liquid nitrogen immediately after resection and stored at −80°C until use.

### Cell culture, treatment and X-irradiation

Cells were grown on cover slips in a humidified 5% CO_2_ atmosphere at 37°C in DMEM supplemented with 10% fetal bovine serum and 1% antibiotics (10,000 U/ml penicillin and 10,000 *μ*g/ml streptomycin). Cells were irradiated with X-rays in the exponential growth phase at room temperature in cell culture medium. All experiments were performed by irradiating cell cultures with 95 kV and 25 mA, with a 1.3-mm aluminium filter and a dose of 1 Gy/min (as determined by chemical dosimetry). NU7026 (KuDos Pharmaceuticals, Cambridge, UK) (10 *μ*M) was added 1 h prior to irradiation and maintained in the medium post irradiation.

### Transfection

For transfection, 2 *μ*g plasmid-DNA per well was used. Plasmid-DNA was complexed at a ratio of 2 *μ*g DNA/5 *μ*l Lipofectamine 2000 (Invitrogen, Carlsbad, CA, USA). Plasmid-DNA and Lipofectamine 2000 were diluted in OPTI-MEM medium (Gibco) and incubated for 5 min at room temperature, followed by complexing and incubation for 20 min. No serum and antibiotics were used in the transfection mixture. After transfection cells were stored at 37°C and 5% CO_2_ for 24 or 48 h.

### siRNA

The Silencer^®^ pre-designed siRNA (Ambion) used for silencing the KUB3 expression consisted of 21 bp duplexes complementary to a unique sequence of the target gene. KUB3-siRNA (CCUUAGUGGAGACUGCUCA) showed an effective silencing in KUB3 expression in TX3868 cells. As a negative control, a non-silencing siRNA duplex (UUCUCCGA ACGUGUCACGUdTdT), fluorescent (3′-fluorescein-conjugated) and non-fluorescent was used (Qiagen). Transfections of cells with these siRNAs were done by using Lipofectamine 2000 (Invitrogen, Carlsbad, CA, USA) at a ratio of 1.3 *μ*g siRNA/6.5 *μ*l Lipofectamine 2000.

### Antibodies

A rabbit anti-KUB3 antibody was raised against KUB3 N-terminus (DERRRGPAAGEQLQQQHVS) and was used at a 1:100 dilution for western blot analysis. The following antibodies were used at the indicated concentration: mouse monoclonal anti-γ-H2AX antibody (Upstate Biotechnology) at a 1:200 dilution for immunofluorescence; mouse monoclonal anti-β-actin (clone AC-15) (Sigma) at a 1:10,000 dilution for western blotting. Horseradish peroxidase-conjugated secondary antibodies were purchased from Jackson ImmunoResearch Laboratories (Dianova): goat anti-rabbit IgG (dilution 1:20,000) and sheep anti-mouse IgG (dilution 1:30,000). The fluorescent secondary antibody Alexa Fluor 488-conjugated goat anti-mouse (MoBiTec) was used at a 1:500 dilution.

### Immunofluorescence

Cells grown on cover slips were fixed in 100% methanol (−20°C) for 30 min, permeabilized in acetone (−20°C) for 1 min and washed three times for 10 min in 1% FCS/PBS. Samples were incubated with primary antibody in 1% FCS/PBS at room temperature for 1 h, washed in 1% FCS/PBS three times for 10 min and incubated with secondary antibody at room temperature for 1 h. Cells were washed in PBS four times for 10 min and mounted using Vectashield mounting medium containing 4,6-diaminodino-2-phenylindole (Vector Laboratories). In a single experiment, cell counting was performed until ≥40 cells and 40 foci were registered per sample. Each data-point represents three independent experiments. Error bars represent the standard error of the mean (SEM) between the different experiments.

### Tumor cell lysates

Approximately 0.5 g tumor tissue was homogenized in 2.5 ml sample buffer [5.8% SDS (w/v), 120 mM Tris pH 6.8; 9.5% β-mercaptoethanol (v/v), 9.5% glycerol (v/v)] supplemented with protease inhibitor cocktail (Complete Mini EDTA free, Roche). After an incubation time of 10 min at room temperature the supernatant was collected by centrifuging at 3,000 g at 4°C for 20 min. This solution was homogenized and centrifuged at 3,000 g at 4°C for 20 min. The samples were boiled at 98°C for 5 min and then centrifuged at 12,000 g at 4°C for 30 min to remove any precipitate.

### Western blotting

Whole cell extracts of treated or mock-treated cells were obtained by direct lysis in lysis buffer (50 mM Tris-HCl pH 8.0, 150 mM NaCl, 1% NP40) supplemented with protease inhibitor cocktail (Complete Mini EDTA-free, Roche). Concentrated loading sample buffer was added for 1X final concentration in all fractions and the samples were boiled for 5 min. Equal aliquots of each sample were separated on SDS-PAGE (12%) and blotted onto Hybond-P membrane (Amersham). Human brain whole cell lysate (Abcam) was used as control. Membranes were blocked with Tris-buffered saline (TBS) containing 5% skim milk and 0.05% Tween-20 (Sigma) at 4°C overnight. Immunostaining was performed in the same buffer using appropriate first and secondary antibodies. Protein detection was performed using the ECL Plus detection kit (Amersham) according to the manufacturer’s instructions.

## Results

**Figure 1.**
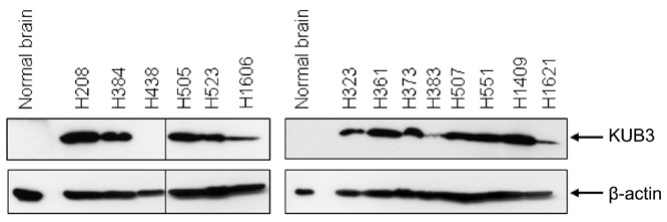
Western blot analysis of endogenous KUB3 protein in different glioblastomas and in normal brain. Total protein extract (30 *μ*g) was separated by SDS-PAGE. The membrane was immunostained by anti-KUB3. β-actin staining confirmed equal loading. Arrows indicate KUB3 at 32 kDa.

*KUB3* is known to be frequently amplified and/or overexpressed as mRNA in GBM, however, no evidence for KUB3 protein overexpression in GBM tissues has been reported. Using western blotting we analyzed cell lysates from 14 GBM tissues to confirm KUB3 overexpression in GBM. We found strong KUB3 protein expression in 10 GBM, weak expression in 3 cases and lack of expression in one GBM and in normal brain that served as negative control (Fig. 1). These western blotting data are in agreement with the frequent mRNA overexpression in GBM. Although overexpression of KUB3 is more frequent than *KUB3* amplification, KUB3 overexpression appears to be mediated not only by DNA amplification but also by other mechanisms. The first hint towards the function of KUB3 stems from Boothmann and colleagues that found binding to Ku70 by yeast-two-hybrid studies. We confirmed this binding by co-immuno precipitation of FLAG-KUB3 and Ku70 protein using COS1 cells (data not shown).

**Figure 2.**
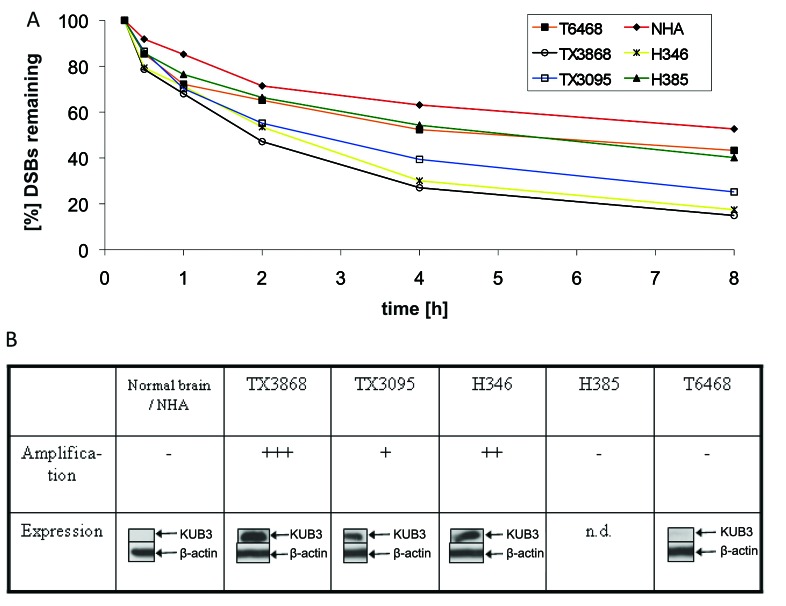
(A) Correlation between KUB3 amplification and DSB repair efficiency in glioma derived cell cultures and normal human astrocytes. Following radiation with 1 Gy, γ-H2AX foci were counted in glioblastoma cell lines TX3095, TX3868, H346 and H385, in astrocytoma (WHO II) T6468 and in normal human astrocytes (NHA). Percentage of remaining γ-H2AX foci (indirect readout for DSBs) is indicated on the y-axis. The repair time is plotted on the x-axis. (B) Expression and amplification status of glioma derived cell lines, normal brain and normal human astrocytes. Total protein extract (30 *μ*g) was separated by SDS-PAGE. The membrane was immunostained with anti-KUB3. β-actin staining confirmed equal loading. Arrows indicate KUB3 at 32 kDa.

Next, we examined whether *KUB3* amplification can be associated with the DSB repair efficiency in GBM. We derived cell cultures from two primary GBMs (H346 and H385), two xenografts (TX3868 and TX3095) and a WHO II astrocytoma (T6468). These selected cell cultures showed different levels of *KUB3* amplification. The amplification level was highest for TX3868, followed by H346 and TX3095. No amplification of *KUB3* was found for H385 and for T6468 (Fig. 2B).

The level of amplification mirrors the level of KUB3 expression as shown by western blotting with a polyclonal KUB3 antibody. Cell cultures with high *KUB3* amplification show strong KUB3 expression whereas low-level *KUB3* amplification was associated with a very weak KUB3 expression (Fig. 2B). Likewise, normal brain that was used as reference for western blotting showed a very weak KUB3 expression. From GBM culture H385 we did not obtain sufficient protein.

Each of the cell cultures was irradiated with 1 Gy. As control we used normal human astrocytes (NHA). Between 15 min and 8 h after radiation, we counted the number of DSBs that were visualized as γ-H2AX foci (Fig. 2A). Throughout this time period low numbers of foci were found in TX3868, H346 and TX3095. These data indicate an efficient double-strand break repair in GBM cell cultures that harbour an amplified *KUB3* gene and show elevated KUB3 protein expression. The most efficient repair was found in the GBM cell cultures with the highest amplification and expression level of KUB3 namely TX3868 and H346. After 8 h following radiation close to 85% of the DSBs were repaired.

Less efficient repair was found in the GBM cell line H385 that showed no amplification and in the astrocytoma WHO II derived cell line T6468 that shows neither amplification nor expression. We found the least efficient DNA repair in normal human astrocytes. DSB repair in TX3868 cells with high *KUB3* amplification and expression is approximately twice as efficient as DSB repair in normal astrocytes that repaired 50% of the DSBs after 8 h. Each experiment was repeated three times. Except for the NHA cells we found very small error bars for each cell culture. The results are indicative of a general correlation between the level of *KUB3* amplification and expression in glioma and the repair efficiency of DSBs (Fig. 2).

**Figure 3.**
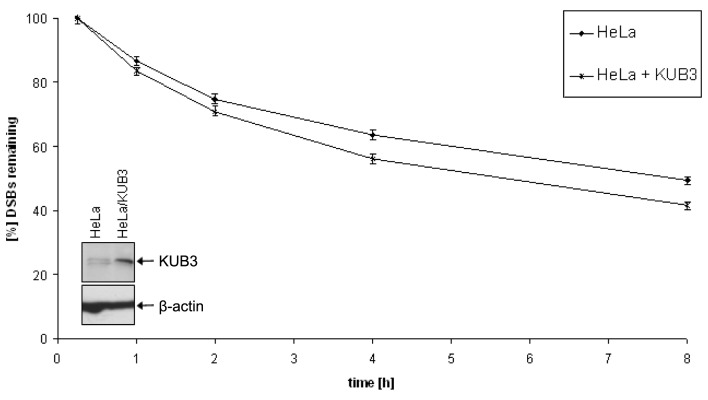
DSB repair efficiency in transfected HeLa cells that overexpress KUB3 and in HeLa cells with low endogenous KUB3 expression. KUB3 expression was demonstrated by immunostaining with anti-KUB3. β-actin staining confirmed equal loading. Following radiation with 1 Gy, γ-H2AX foci were counted in HeLa cells. Percentage of remaining γ-H2AX foci (indirect readout for DSBs) is indicated on the y-axis. The repair time is plotted on the x-axis. Error bars represent the SEM of the analysis of 40 cells in three different experiments.

To provide functional evidence for the observed correlation, we compared untreated HeLa cells with HeLa cells transfected with *KUB3*. While KUB3 was barely visible in untreated HeLa cells, transfected cells showed a KUB3 signal in western blot analysis (Fig. 3). Both transfected and untransfected HeLa cells were irradiated with 1 Gy and analyzed for the number of γ-H2AX foci. HeLa cells transfected with *KUB3* showed less foci indicating a better double-strand break repair than untreated HeLa cells (Fig. 3). As for the analysis of glioma cell cultures, each experiment was repeated three times and the results were highly reproducible. The relatively moderate improvement of the DSB repair efficiency in transfected HeLa cells may be explained by the moderate expression of the KUB3 protein found upon transfection. This expression is considerably lower than the expression found in glioblastoma cells TX3095 and TX3868 that also showed a significantly more efficient repair efficiency of DSBs.

**Figure 4.**
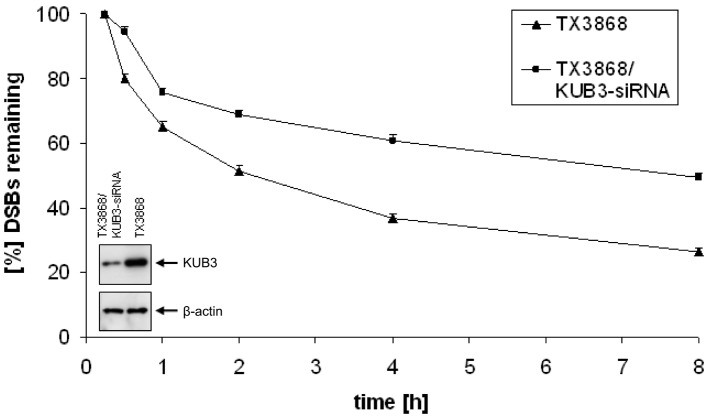
DSB repair efficiency in TX3868 and TX3868 cells transfected with KUB3 siRNA. KUB3 expression was demonstrated by immunostaining with anti-KUB3. β-actin staining confirmed equal loading. Following radiation with 1 Gy, γ-H2AX foci were counted in HeLa cells. Percentage of remaining γ-H2AX foci (indirect readout for DSBs) is indicated on the y-axis. The repair time is plotted on the x-axis. Error bars represent the SEM of the analysis of 40 cells in three different experiments.

To further test the effect of altered KUB3 expression on repair efficiency we reduced the high endogenous KUB3 expression in TX3868 cells by transfecting these cells with KUB3 siRNA. The expression level was reduced significantly as shown in Fig. 4. We also found a significantly increased number of γ-H2AX foci in TX3868 cells treated with KUB3 siRNA in comparison to untreated TX3868 cells (Fig. 4). Four hours after radiation >65% of the DSBs were repaired in untreated TX3868 cells as compared to 40% in TX3868 cells treated by KUB3 siRNA. Similar ratios were found after 8 h with >75% of repaired DSBs in untreated TX3868 cells and 50% in treated TX3868 cells. In summary, both the transfection with *KUB3* and the treatment with KUB3 siRNA demonstrates that altered KUB3 expression impact the repair efficiency.

**Figure 5.**
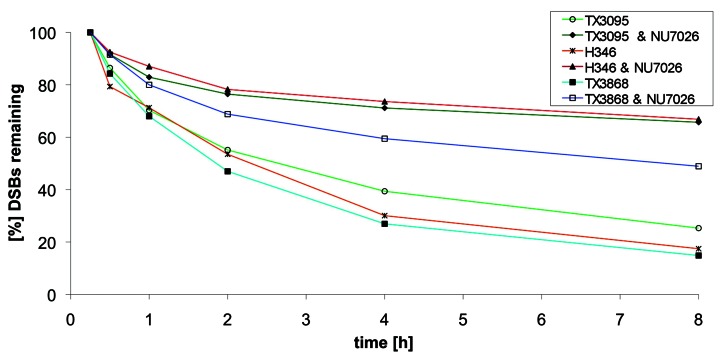
DSB repair efficiency in TX3095, TX3868 and H346 cells analyzed in the presence of DNA-PK inhibitor NU7026. Following radiation with 1 Gy, γ-H2AX foci were counted in NU7026 treated glioblastoma cell lines TX3095, TX3868 and H346. Results from the untreated glioblastoma cell lines were included in this figure for better visualization of the differences between DSB repair with and without NU7026. Percentage of remaining γ-H2AX foci, indirect readout for DSBs is indicated on the y-axis. The repair time is plotted on the x-axis.

Binding of KUB3 to Ku70 suggests that KUB3 exerts its effect on DSB repair via DNA-PK. To clarify whether the observed effects of KUB3 on double-strand repair are dependent or independent of DNA-PK activity, we inhibited DNA-PK activity by NU7026 in the cell cultures with high *KUB3* amplification and expression and with elevated repair efficiency. Specifically, we analyzed TX3095, TX3868 and H346 cells. In all three cell cultures we found reduced double-strand repair efficiency after NU7026 treatment. The extent of reduction was similar in TX3868 and TX3095. TX3868 cells that had the highest repair efficiency prior to inhibition of DNA-PK, showed also the highest repair efficiency after the treatment as compared to the cell cultures TX3095 and H346. TX3095 cells with the lowest repair efficiency maintained this low efficiency after treatment as compared to TX3868. The most significant reduction in repair efficiency was found for H346 (Fig. 5).

## Discussion

Although frequent gene amplifications in glioma are well established, we know little about the specific function of most amplified genes for the tumor phenotype. Here, we show that both the amplification level and the expression level of KUB3 are associated with the efficiency of DSBs in glioblastoma. KUB3 seems to influence DSB repair efficiency via DNA-PK dependent repair pathway.

An increased DSB repair as a result of a *KUB3* amplification may be part of the mechanisms that contribute to the radiation-resistant phenotype frequently found for glioblastoma cells. Circumstantial evidence for such a link can be derived from various studies. A comparison of transcriptional changes in two glioblastoma cell lines with different degrees of radiation-sensitivity identified a larger number of genes known to be involved in DSB repair (18). One of the genes identified as differentially expressed in the two cell lines was *G22P1 (Ku70)*. As shown in a clonogenic assay tumors with a low frequency of Ku70 immunopositive cells are radiosensitive (19).

Besides Ku expression, DNA-PK expression was also related to the radiation-resistance of tumor cells. Overexpression of the catalytic subunit DNA-PKcs was reported for various human cancers (20–24). Inhibition of the function of the DNA-PK sensitized tumor cells to radiation as shown for cervical cancer (25). A study on oral squamous cell carcinoma showed that upregulation of DNA-PK complex protein following radiation treatment correlates to radiation resistance (23). There is functional evidence for a causative role of DNA-PKcs in the radiation-resistant phenotype of glioblastoma. While glioblastoma cell line MO59J that lacks DNA-PKcs is radiosensitive, transfection of DNA-PKcs in MO59J reverses the radiation-sensitive phenotype (26,27). Inhibition of the DNA-PK expression also enhances the sensitivity of cells to chemotherapeutic agents (28).

One has, however, to be cautious to prematurely propose a simple link between radiation-resistance and double-strand break repair efficiency. Recent data demonstrate inefficient repair of double-strand breaks in several radiation-resistant glioblastoma cell lines (29). Such an inefficient repair is possible due to the genetic background of a tumor, specifically due to TP53 mutations that are frequently found in glioblastoma (30).

In addition to the radiation-resistance of tumor cells, patients’ overall survival has been associated with DSB repair. Tumors with a low percentage of Ku70 positive cells have been associated with a significantly higher overall patients’ survival. This association was also found for Ku80, but did not reach significance. In addition, expression of Ku70 was identified as possible prognostic factor for the survival for cervix carcinoma patients undergoing radiotherapy (19). Strong evidence for an association between DNA repair and survival of glioma patients stems from studies on DNA repair enzyme O6-methyl-guanine DNA methyltransferase (MGMT) and the alkylating agent temozolomide (TMZ). MGMT that is a central enzyme for DNA repair, removes mutagenic adducts from O6-guanine in DNA. Silencing MGMT by methylating its promoter, has been associated with improved survival rates of glioblastoma patients that were treated with TMZ (31,32). Likewise, downregulation of MGMT by interleukin-24 helps to overcome TMZ resistance (33). By contrast, MGMT expression predicts a shorter overall survival of glioblastoma patients treated by TMZ (31). This observation is in good agreement with the observation that glioma patients without *KUB3* amplification show a prolonged survival in comparison to patients with *KUB3* amplification (15).

While KUB3 is involved in DSB repair, it does not seem to play a role in the V(D)J-joining (data not shown). Other proteins of DNA-PK, including DNA-PKcs, Ku70/80 heterodimer, XRCC4, ligase 4, artemis and cernunnos-XLF, all of which known be involved in non-homologous end joining were also found to be involved in the V(D)J recombination (13,14,34–42). The lack of any of these proteins results in a SCID phenotype due to the inability to generate V(D)J joins (43,44). Since our preliminary results do not indicate an involvement of KUB3 in V(D)J recombination, interaction of KUB3 with DNA-PK may be different from the known interactions of the aforementioned repair genes. Possibly, KUB3 is not part of the DNA-PK complex but may only exert a regulatory function.

In conclusion, we propose that *KUB3* amplification contributes to the radiation-resistant phenotype of glioblastoma and impact survival of glioblastoma patients. While our previous study demonstrated an association between *KUB3* amplification and shortened survival of glioblastoma patients and this study analyzed the role of KUB3 in DSB repair, future studies are required to establish a role of KUB3 for the radiation-resistant phenotype of glioblastoma cells.
